# 523. Efficacy and Safety of Nirmatrelvir/Ritonavir for COVID-19 in Adolescents

**DOI:** 10.1093/ofid/ofad500.592

**Published:** 2023-11-27

**Authors:** Kiriam Escobar Lee, Philip J Lee, Brenda I Anosike

**Affiliations:** Children’s Hospital at Montefiore, Albert Einstein College of Medicine, Bronx, New York; Children's Hospital at Montefiore, Bronx, New York; Children's Hospital at Montefiore, Bronx, New York

## Abstract

**Background:**

Nirmatrelvir/ritonavir (n/r) is a currently 1 of 2 oral medications FDA-approved for treatment of COVID-19 in patients ≥ 12 years of age AND ≥ 40 kg. To date, dosing recommendations for adolescents are extrapolated primarily from adult studies. We conducted a retrospective study at a large pediatric academic center to evaluate the efficacy and safety of n/r in adolescent patients.

**Methods:**

Retrospective electronic chart review of adolescents ages 12-18 years diagnosed with COVID-19 between 1/1/2022 to 3/31/2022. We included those with laboratory confirmed COVID-19 by either PCR or rapid antigen, treated with n/r within 5 days of symptom onset. The primary endpoint was COVID-related hospital admissions, ER and/or outpatient visits within 30 days after completion of n/r. Our secondary endpoint was COVID-related adverse event(s) due to n/r.

**Results:**

A total of 91 unique patients were prescribed n/r therapy; 1 was prescribed twice. All but 2 were ordered in the outpatient setting. 34.1% (29/85) had a BMI ≥ 30 kg/m2. Thirty-eight (41.8%) and 25 (27.5%) patients received 2 and 3 COVID-19 vaccines, respectively. Asthma (26/91, 28.6%) was the most common underlying condition; (16/91, 16.6%) were immunocompromised. Median time from initial COVID-19 positivity to n/r prescription was 0 days (range: 0-4). 2.2 % (2/90) of those who received outpatient prescriptions returned to the ER with COVID-19 like symptoms within 30 days. Another 2 different patients who were prescribed n/r inpatient expired at 28 and 48 days after completion of therapy. Only 2.2% (2) experienced an adverse event after completion of n/r. (Table 1)

**Figure:**
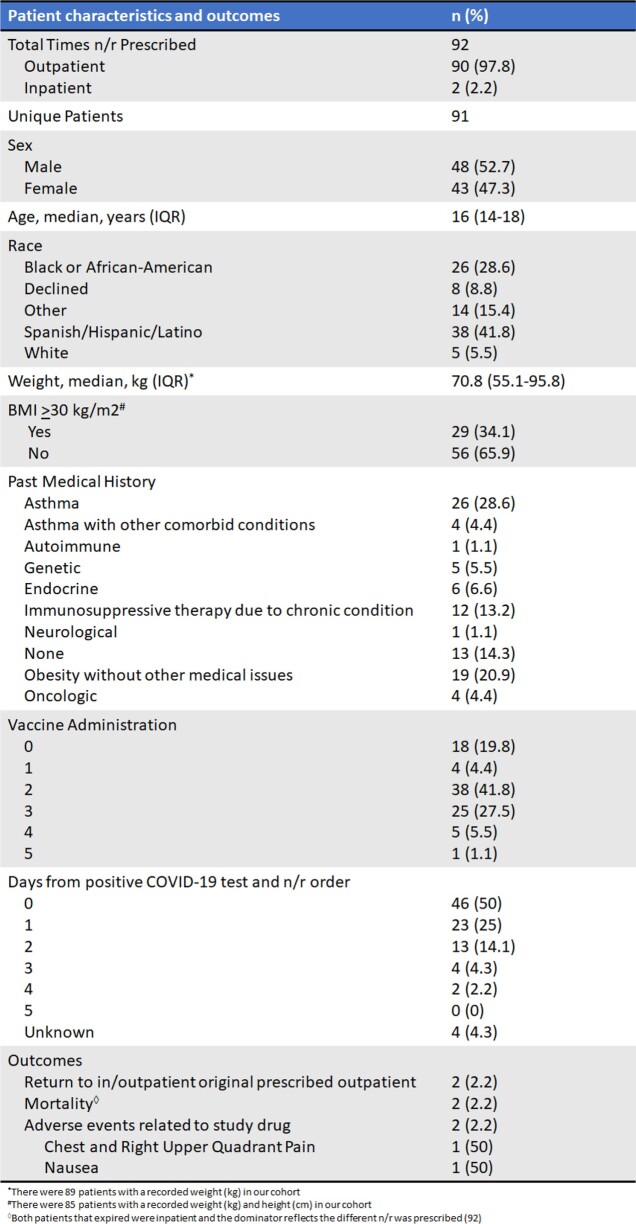

Demographic and outcome information of adolescent patients treated with nirmatrelvir/ritonavir for COVID-19 infection at a large pediatric academic medical center.

**Conclusion:**

Though limited, this single center study demonstrated low rates of COVID-related hospital admissions, ER and/or other outpatient visits as well as adverse events suggesting that n/r may be a safe and well tolerated by most adolescents diagnosed with COVID-19. Larger/prospective pediatric studies are still needed.

**Disclosures:**

**All Authors**: No reported disclosures

